# Machine learning pipeline to analyze clinical and proteomics data: experiences on a prostate cancer case

**DOI:** 10.1186/s12911-024-02491-6

**Published:** 2024-04-08

**Authors:** Patrizia Vizza, Federica Aracri, Pietro Hiram Guzzi, Marco Gaspari, Pierangelo Veltri, Giuseppe Tradigo

**Affiliations:** 1 Department of Surgical and Medical Sciences, Magna Græcia University, 88100 Catanzaro, Italy; 2 Department of Experimental and Clinical Medicine, Magna Græcia University, 88100 Catanzaro, Italy; 3 Department of Computers, Modeling, Electronics and Systems Engineering, University of Calabria, 87036 Rende, Italy; 4 Department of Theoretical and Applied Sciences, eCampus University, 22060 Novedrate, CO Italy

**Keywords:** Machine learning, Prostate cancer, Biological pipeline, Data enhancing

## Abstract

Proteomic-based analysis is used to identify biomarkers in blood samples and tissues. Data produced by devices such as mass spectrometry requires platforms to identify and quantify proteins (or peptides). Clinical information can be related to mass spectrometry data to identify diseases at an early stage. Machine learning techniques can be used to support physicians and biologists in studying and classifying pathologies. We present the application of machine learning techniques to define a pipeline aimed at studying and classifying proteomics data enriched using clinical information. The pipeline allows users to relate established blood biomarkers with clinical parameters and proteomics data. The proposed pipeline entails three main phases: (i) feature selection, (ii) models training, and (iii) models ensembling. We report the experience of applying such a pipeline to prostate-related diseases. Models have been trained on several biological datasets. We report experimental results about two datasets that result from the integration of clinical and mass spectrometry-based data in the contexts of serum and urine analysis. The pipeline receives input data from blood analytes, tissue samples, proteomic analysis, and urine biomarkers. It then trains different models for feature selection, classification and voting. The presented pipeline has been applied on two datasets obtained in a 2 years research project which aimed to extract hidden information from mass spectrometry, serum, and urine samples from hundreds of patients. We report results on analyzing prostate datasets serum with 143 samples, including 79 PCa and 84 BPH patients, and an urine dataset with 121 samples, including 67 PCa and 54 BPH patients. As results pipeline allowed to identify interesting peptides in the two datasets, 6 for the first one and 2 for the second one. The best model for both serum (AUC=0.87, Accuracy=0.83, F1=0.81, Sensitivity=0.84, Specificity=0.81) and urine (AUC=0.88, Accuracy=0.83, F1=0.83, Sensitivity=0.85, Specificity=0.80) datasets showed good predictive performances. We made the pipeline code available on GitHub and we are confident that it will be successfully adopted in similar clinical setups.

## Introduction

**Fig. 1:**
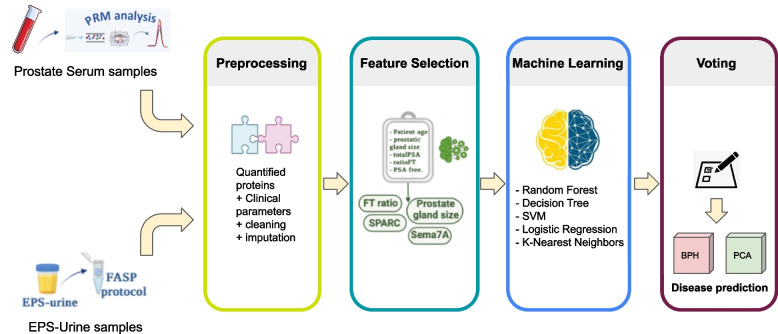
Figure reports the pipeline workflow, consisting of (from left side): (i) Prostate serum and EPS-urine datasets; (ii) the preprocessing phase, allowing to remove the inconsistent values and to correct the missing values; (iii) the feature selection phase allows us to keep only the most important features to improve the application of the classification algorithms; (iv) the ML phase, consisting in choosing among five different classification algorithms; (v) finally, the voting phase, which consists of a soft vote and hard vote, to support disease prediction using two unknown datasets (one for serum and one for urine respectively)

Machine learning and artificial intelligence-based techniques can be used to analyze cancer-related data to support clinicians in biomarker identification. For instance, statistical methods such as principal component analysis, as well as hierarchical clustering analysis can be used to identify lipid molecules for prostate cancer diagnosis [1]. We designed and developed a pipeline, available on GitHub^1^, to analyze a large biological dataset (e.g. mass spectrometry data) enriched by clinical information. The pipeline aims to discover novel biomarkers by using machine learning models. The pipeline, whose architecture is reported in Fig. 1, also uses a voting mechanism to obtain the best overall predictions. The architecture includes a mechanism to track experimental processes relating biological data with clinical ones. Data for the pipeline has been collected from an information system that can gather clinical and biological data [2], which tracks information about clinical and biological samples collected from health structures and biological laboratories (e.g., MS laboratories). The tracking system contains MS data regarding tissues and blood samples from patients affected by cancer-related conditions. The pipeline includes LR (Logistic Regression), DT (Decision Tree), KNN (K-Nearest Neighbors), SVM (Support Vector Machine) and RF (Random Forest) machine learning models.

We present the structure and the results of applying the developed pipeline to a real case. We report the results of the application on mass spectrometry data enriched with clinical information regarding prostate cancer. Prostate cancer (PCa) is one of the most commonly diagnosed types of cancer [3]. Clinical detection uses Prostate-Specific Antigen (PSA) blood-based indicator as screening or diagnostic tests for PCa. Then, PCa diagnosis is based on clinical analysis and ultrasound-guided transrectal biopsy. Clinical data used to enrich the proteomics analysis regarded prostate gland dimension and gland biopsy results (e.g., neoplasms types). Clinical data obtained from urology department units (e.g., urine samples, serum data, prostate gland information) have been integrated with biological data in a unique framework. The tracking system included in the pipeline has been used to relate clinical samples with proteomics analysis.

We show how using the proposed pipeline it is possible to support clinicians in decisions and strategies. In the presented example, we started from clinical consideration regarding the fact that prostate biopsy has an appreciable false negative rate, and thus applying the pipeline may support in guiding to reduce the recurrent use of biopsy. A key issue is the definition of the optimal frequency for re-biopsy in men who have had a prior negative biopsy based on PSA level, age, and other factors [4]. The pipeline applied to the prostate cancer dataset, allowed both early detection and reducing the biopsy rate (allowing the reduction of the interventions). The integrated analysis of clinical and biochemical data of patients could lead to the determination of novel biomarkers to monitor the disease. Figure 1 shows an instance of the proposed pipeline on proteomics data and clinical data associated with the prostate cancer disease dataset. Patient data are gathered and used as input to the machine learning models to support physicians in making clinical decisions.

The pipeline has been tested on two clinical datasets, regarding Prostate Cancer (PCa) and Benign Prostatic Hyperplasia (BPH), describing patient prostate conditions which have been enriched with Mass Spectrometry data resulting from analyzing clinical samples. The aim of using the pipeline is to identify a subset of peptides (from blood serum or urine samples), representing biological markers significantly correlating with the presence or absence of prostate cancer [5, 6].

The two biological use cases adopted here to describe the usage of the presented pipeline in real-world scenarios are both binary classification problems (i.e. classes being PCa and BPH). Nevertheless all of the pipeline components, in the feature selection, machine learning, and voting compartments, do support multiclass classification scenarios with minimal adaptation, since all of the adopted models can predict more than two classes. As we report in the experimental results, the application of the pipeline in the two used datasets, allowed users to reduce the total number of features for a multivariate test, from 37 to 11 features for the Prostate Serum dataset, and from 1677 to 4 features for the EPS-Urine dataset (e.g., semaphorin-7A, secreted protein acidic and rich in cysteine (SPARC), FT ratio, Prostate Gland Size).

Furthermore, the pipeline is capable of working with non-categorical (i.e. continuous) variables, by selecting a different set of ML models able to deal with continuous clinical variables (e.g. regressors, neural networks, support vector machines).

The use of machine learning techniques to identify biomarkers has been widely adopted for knowledge extraction from biological as well as clinical data [7–10]. The aim is to support physicians and biologists in identifying relevant biomarkers that can help in characterizing diseases. Moreover, many papers in the literature discuss about the use of knowledge extraction boosting and results enrichment with the help of integrated models using voting mechanisms. In [11] ML models have been used for data reduction in mass spectrometry datasets. In [12] and in [13–15] ML techniques are used to select biomarkers from prostate and ovarian cancer datasets respectively. Voting has been used in [16, 17] to improve protein identification in different use cases.

The use of machine learning-based techniques for prostate cancer data analysis is widely represented in the literature. For instance in [12, 13, 15] ML techniques have been used to select significant biomarkers in prostate datasets. Specifically, in [15], the authors used mass spectrometry data analysis to discover and validate prostate-derived proteins in fluids.

In [18] machine learning-based models have been used to assess the potential role of the inflammation biomarkers in the prediction of myocardial infarction. The proposed approach is based on a set of interpretable rules supported by clinical evidence and selected for a given patient by using a machine learning classifier to estimate cardiovascular risk. Battista et al. in [19] proposed a predictive system based on serum biomarkers and ensemble learning to predict colorectal cancer presence and its stage. Authors in [20] used machine learning to identify the optimal diagnostic biomarkers for non-small cell lung cancer by using least absolute shrinkage, selection operator logistic regression, support vector machine, and recursive feature elimination. In [21] a hybrid machine learning systems strategy has been proposed to obtain a transcriptome profile linked with classification procedures aiming to support the early detection of breast cancer. This strategy includes feature selection algorithms, a feature extraction algorithm, and classifiers for diagnosing breast cancer. Authors in [22] presented a non-invasive breast cancer classification system for the diagnosis of cancer metastases based on machine learning models extracting information from blood profile data. This system may assist physicians in selecting intensive care for patients with metastatic breast cancer to enhance the overall survival outcome.

The use of ML-based prediction tools in biological pipelines is present in the literature. In [23] authors treat early detection of type 2 diabetes mellitus using machine learning-based prediction models. Pattern recognition, disease prediction, and classification using various data mining techniques have been used to analyze biomedical datasets [24, 25].

The here proposed pipeline uses ML models to support physicians and biologists in studying peptides and biomarkers extracted during biological experiments, integrated via voting mechanisms [26]. Experimental results have been useful for biological and clinical interpretation [27, 28] in the context of early predictions of diseases.

The paper is organized as follows. In “Methods” section we describe the methods and tools used to design the pipeline’s modules for the preprocessing, feature selection and training of the machine learning models used for the classification task. In “Results” section we present the application of the pipeline to process two datasets and the prediction results obtained by the trained machine learning models and finally give some details about the system implementation. In “Discussion” section we explore and discuss the implications of the results of the work.

## Methods

The pipeline architecture is reported in Fig. 1. It is composed by the following modules: (*i*) Data acquisition, which acquires data from databases and tracking systems (i.e., the mechanisms able to track samples that are analyzed and treated in different laboratories [2]); (*ii*) Preprocessing module, in charge of performing data preparation; (*iii*) Feature selection, which is in charge of identifying interesting features; (*iv*) ML models training, which is in charge of selecting machine learning models; (*v*) Voting module, used to support models selection. The pipeline has been implemented by using the Jupyter programming environment [29], which allows the user to write an interactive notebook containing executable scientific experiments in the Python programming language. We report in the following the principle phases, starting from preprocessing one.

### Preprocessing phase

We performed data preprocessing by taking into consideration: (i) missing values, (ii) values expressed in different scales or measurement units, (iii) null values, (iv) outlier values. In case of input file with missing values, represented by *NaN* (Not a Number) literals, corresponding records are dropped in case of large number, otherwise the missing value are corrected by using the average of all values. For instance, for the PCa and BPH we calculated the average of each subset and we replaced the mean value of the subset corresponding to the missing value in the Prostate Gland Size column. Similarly, the missing values were inserted: age of patient, PSA free, PSA ratio, total PSA, and some proteins. Also, at the preprocessing step, the pipeline eliminates the columns with data considered irrelevant (e.g., the columns containing information about previous surgery and previous prostate biopsy). Moreover, a normalization function has been applied by converting the categorical values into numeric classes and normalizing all numeric values in the range from 0 to 1.

### Feature selection

The pipeline feature selection module (see “Feature Selection” box in Fig. 1) implements the following models: (*i*) Pearson correlation coefficient [30], (*ii*) Chi-Square Test [31], (*iii*) RFE (Recursive Feature Elimination) [32], (*iv*) Random Forest [33] and (*v*) Logistic Regression [34]. The feature selection module has been implemented in Python (see first part of Algorithm 1).

**Figure:**
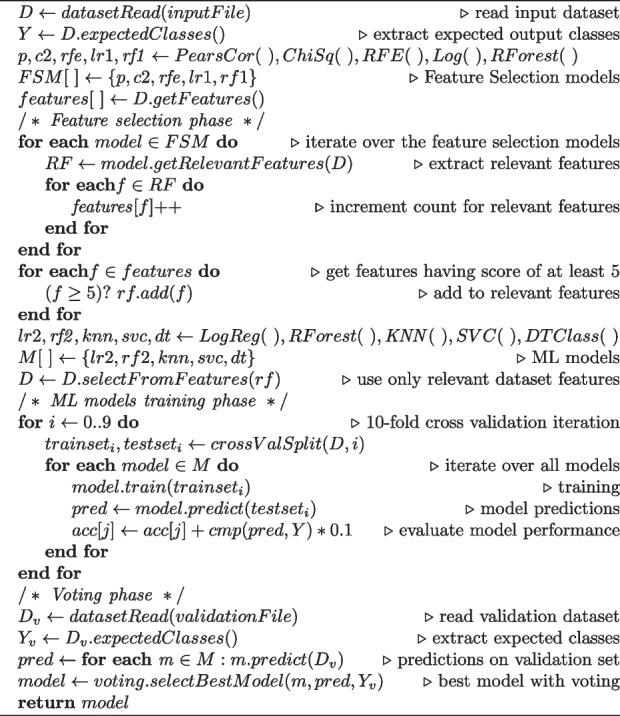
**Algorithm 1** Feature Selection and Machine Learning phases of the pipeline

Feature selection identifies the most statistically significant features (i.e. columns of the dataset) according to each model’s metrics, and ranks them according to a relevance score (i.e. how many models agreed on its relevance). After evaluating different features (e.g. considering the ones with a total consensus of 5 models, considering the ones higher than 4, etc.) the pipeline selects the best predictive performances achieved by considering all models. Pearson correlation coefficient [35] measures the linear correlation between two features. Let X and Y be a pair of random variables, the Pearson correlation coefficient is the ratio between their covariance and the product of the standard deviations of the two variables. The relationship between the correlation coefficient matrix, *R*, and the covariance matrix, *C*, built from X and Y values (in our case two of the features), is $R_{i,j} = C_{i,j} / \sqrt{C_{i,i} C_{j,j}}$. The values of R are between -1 and 1, inclusive. The correlation coefficient formula is used to find relationships between couples of features. It returns continuous values between -1 and 1, where 1 indicates a full correlation (total positive linear correlation), while -1 indicates a missing correlation (total negative linear correlation) between the two features (values near 0 indicate no correlation). At the end of the process, only features with low Pearson correlation coefficients are kept. Moreover, the pipeline includes Chi-square which here is used to test the independence of two features [36]. Given two variables, the test measures how the observed count and expected count deviate from each other. When two variables are independent, the observed count is close to the expected count, resulting in a smaller Chi-square value (high Chi-square values indicate that the hypothesis of independence is incorrect).

We then include, in the feature selection module, the Recursive Feature Elimination (RFE) [37], to fit the model and remove the weakest features. The RFE allows the user to reduce existing colinearity in input data by recursively eliminating features. In a nutshell, RFE allows the user to recursively prune features by looking at data, which represent their relative relevance.

Random Forest (RF) ensures good data abstraction results also because it is easy to calculate the relative importance of each feature on the generated decision tree [38]. RF generates a number (often hundreds) of random decision trees, consisting of a set of nodes with binary questions based on a single or combination of features. At each node, the tree divides the dataset into 2 subsets. The importance of each feature (or set of features) is then calculated by considering how well the feature splits (hence describes) the dataset. Finally, the feature selection module (Fig. 1) includes Logistic Regression (LR), which is a method to remove redundant features from a dataset.

### Machine learning

The pipeline architecture (see “Machine Learning” box in Fig. 1) includes a module training the Machine Learning models, which includes: Logistic Regression (LR), Support Vector Machine (SVM), Decision Tree Classifier (DTC), K-Nearest Neighbors (KNN) and Random Forest (RF) [39].

In order to evaluate the performance of the ML models and minimizing the bias introduced by a simplistic data splitting into training and test sets, we adopted an k-fold cross-validation method. In particular we chose the 10-fold cross-validation approach since evidence shows that k=10 is the best number of splits on average in a large number of experiments, hence it is widely adopted by the scientific community. Logistic Regression (LR) is chosen for its ability to generalize a multivariate dataset. Decision Tree Classifier (DTC) is used both as a predictive model or as a guide to conclude a set of observations. K-Nearest Neighbors (KNN) uses a distance function where instances are assigned to a class according to the most common class among its nearest neighbors. Support Vector Machine (SVM), widely used to solve classification and regression problems in bioinformatics and computational biology, uses a cost parameter for measuring misclassification during data training and a Gaussian radial basis function Gamma ($\gamma$). Random Forest (RF) is used for its efficiency in estimating the relative importance of features.

The ML process steps is reported in the second part of Algorithm 1.

Evaluation is performed based on the following measures: (*i*) AUC (Area Under the Curve), (*ii*) Accuracy, (*iii*) F1-score, (*iv*) Sensitivity and (*v*) Specificity.

### Voting

After the training phase, we performed a model ensemble phase in which different models were integrated in order to increase the accuracy of the prediction (see “Voting” box in Fig. 1). The motivation for such a phase is that the ensemble model will show better prediction performances on average with respect to each single model from which it is composed. We adopted two voting mechanisms, based on the following strategies: (*i*) *hard voting*, which considers the count of models which are in agreement on the classes predicted by each model as a majority consensus; and (*ii*) *soft voting*, in which model predictions are weighted based on the predictive accuracy achieved by each model on the test set.

Without a lack of generality, an instance of the pipeline is reported in Fig. 1 for a prostate cancer dataset. It performs the steps for the general purpose architecture reported above, i.e., *i* preprocessing; *ii* feature selection; *iii* model training phase and assessment; *iv* ensembling of the models through soft and hard voting.

## Results

The proposed pipeline has been implemented and it is available for general purpose ML-based analysis of clinical and biological data. We tested the proposed pipeline on prostate cancer datasets obtained during a 2 years research project called INNOPROST, involving companies, research centers, and a clinical focused on prostate cancer analysis. During the two-year projects, several datasets have been tested employing the proteomics laboratory of the University of Catanzaro, as well as a computer science-based platform aiming to analyze biomedical data. Moreover, datasets imported from a tracking database coupling clinical samples and biological samples processed by the MS analysis laboratory at Magna Græcia University, were analyzed to relate data and peptides as possible biomarkers in prostate cancer diagnosis.

**Fig. 2:**
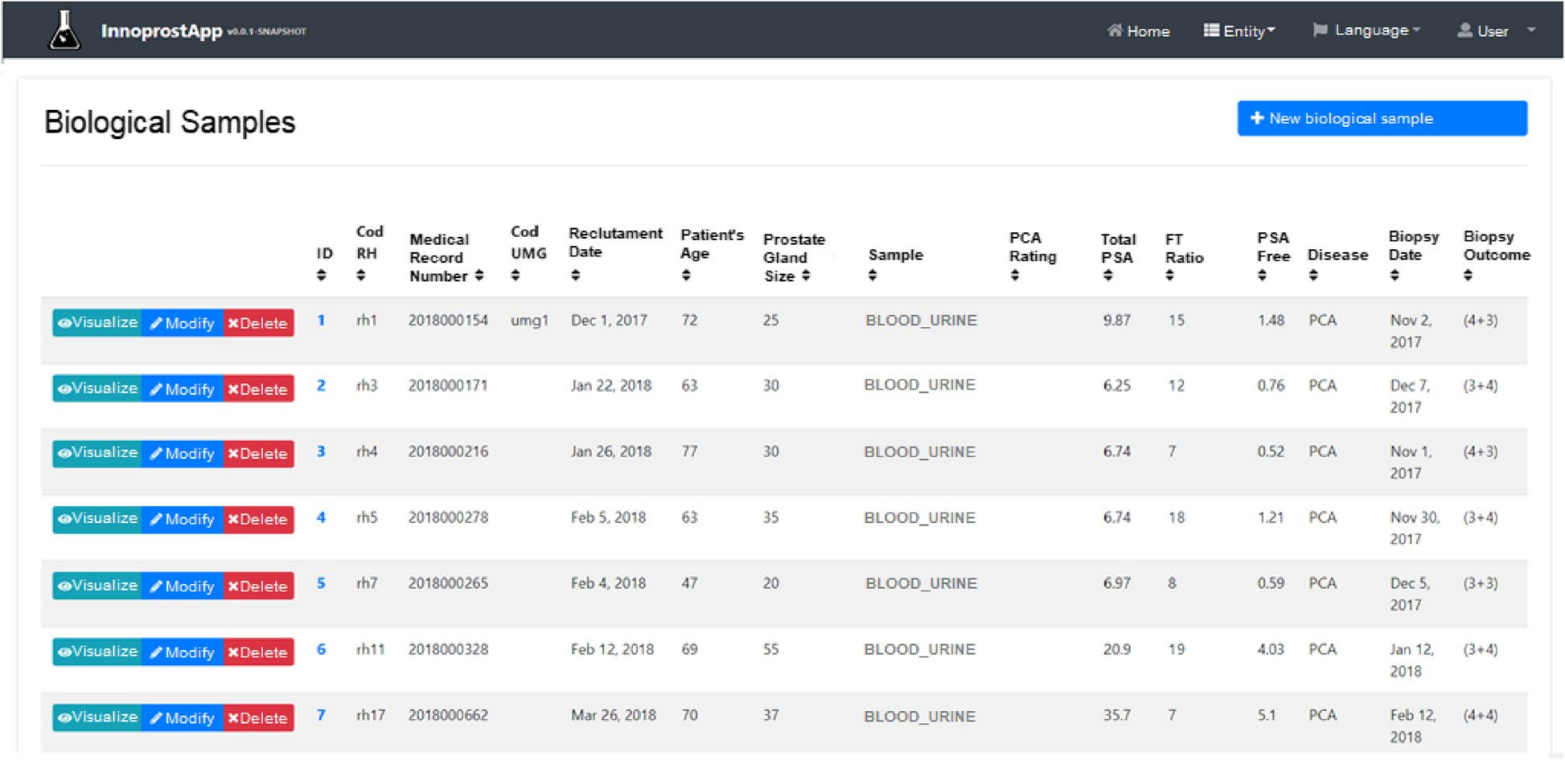
User interface of the sample data tracking. The view shows a list of biological samples and their features (e.g. medical record, recruitment date, age, size of prostate gland)

Figure 2 reports the web-based graphical interface of the system used to store the biological samples, which has been queried to extract the experimental datasets. In prostate cancer analysis, Fig. 2 shows biological information with a set of features: e.g. Medical Record Number, Recruitment Date, Age of patient, and Prostate Gland Size. *Sample* column reports the type of biological sample: it can be *blood*, *urine* or both (i.e. $blood\_urine$ column). *Biopsy Outcome* column expresses the Gleason score of the histology exam.

We used the following two datasets to perform experimental results:

1. Prostate Serum dataset - in which the combination of mass spectrometry data from clinical serum and prostate information are analyzed;
2. EPS-Urine dataset - in which mass spectrometry data from EPS-Urine proteins are analyzed after clinical analysis.

Both above reported datasets have been used to identify features and thus distinguish PCa from BPH. The first dataset contains a total of 143 samples, including 69 patients affected by PCa and 74 by BPH. The 143 samples have been divided according to the 10-fold cross-validation approach into 10 subgroups and the model accuracies (e.g. F1, AUC), collected at each, iteration have been averaged at the end of the cycles.

The second dataset contains a total of 121 patients, including 67 patients affected by PCa and 54 by BPH. The 121 samples have been divided according to the 10-fold cross-validation approach into 10 subgroups and the model accuracies (e.g., F1, AUC), collected at each iteration, have been averaged at the end of the cycles.

Clinical information was extracted from the data tracking clinical information system described above. Table 1 reports the statistics of the main features including age, the size of the prostate gland (expressed as volume in *ml*) obtained by trans-rectal prostate ultrasound, the value of Total PSA and Free PSA (both expressed in *mg/l*), and the ratio between Total and Free PSA (F/T Ratio). For each patient, a set of 1670 peptides was processed. Once data was acquired, the pipeline carried out the following steps, as described in “Methods” section: (*i*) preprocessing; (*ii*) feature selection; (*iii*) ML models training; and (*iv*) voting.

Data preprocessing tackled missing values for the gland size imputing them by using the average value of the corresponding dataset (i.e. Prostate serum and EPS-Urine). Similarly, missing values for the age of the patient, PSA free, PSA ratio, and total PSA have been treated. Also, at the preprocessing step, the pipeline eliminates the columns with too many missing values or data considered irrelevant (e.g., the columns containing information about previous surgery and previous prostate biopsy).

The feature selection phase for the Prostate Serum dataset consisted of selecting features having the highest score (i.e. score of 5). For instance, ProPsa indicates the prostate PSA concentration, the Prostate Gland Size value indicates the prostate gland dimension and 6 peptides referring to relevant proteins were included in the results. Similarly, for the EPS-Urine experiment, features with a score of 5 were adopted, which gave results regarding clinical information (e.g., Protein Gland Size) or proteins (e.g., Semaphorin-7A). In particular, the following peptides or groups of peptides were taken into consideration by domain experts as potentially relevant: (i) Prostate Serum dataset - VQPFNVTQGK (LAMP2), NINYTER, LSDTTSQSNSTAK (LAMB1), LHINHNNLTESVGPLPK (LUM), DGQLLPSSNYSNIK (NCAM1), DFEDLYTPVDGSIVIVR (TFRC); (ii) EPS-Urine dataset - Semaphorin-7A, SPARC.

**Table 1:** Statistic differences between the two classes (PCa and BPH) for the clinical features

	Age		ProstateGlandSize		Total PSA		Free PSA		F/T Ratio	
	PCa	BPH	PCa	BPH	PCa	BPH	PCa	BPH	PCa	BPH
*mean*	66	69	39.78	71.67	10.33	4.02	18.41	39.22	1.73	1.49
*std*	6.23	6.49	14.26	35.86	11.47	5.09	10.88	19.83	1.36	1.95
*min*	47	56	20.00	30.00	3.01	0.07	1.00	0.10	0.52	0.05
*25%*	63	66	30.00	50.00	6.11	0.91	14.00	23.50	0.98	0.20
*50%*	67	71	36.00	66.50	6.75	2.73	16.00	40.00	1.21	0.93
*75%*	72	73	48.25	83.25	8.35	4.49	21.00	54.50	1.68	2.10
*max*	77	81	75.00	173.00	58.40	21.86	62.00	79.00	5.65	9.43

Following the pipeline phases, the ML models training and assessment on both datasets were performed. In the first experimental scenario (Prostate serum), after training, the best performing model was Random Forest [27, 28]. According to the domain experts, the best performing model was chosen by considering the AUC (Area Under Curve) measure. As an example, the Random Forest model was able to discriminate between PCa and BPH with an AUC of 0.87 and F1 of 0.81 [27]. Also, in this case, RF shows high scores in terms of Accuracy and Sensitivity. In general, they are much less likely to overfit than other models since they are composed of many weak classifiers, which are trained independently on completely different subsets of the training data, which ensures low overfitting tendencies.

The proposed computational pipeline has been tested using both serum and urine datasets, through an assessment of performance metrics. Assessment of the Machine Learning algorithms in terms of precision measures for EPS-Urine and Serum datasets allowed us to select the best model and to rank models in terms of various precision metrics: AUC, F1, Accuracy, Specificity, and Sensitivity. In particular, for the cited experiments, Logistic regression, Decision Tree, KNN, SVM and Random Forest, models have been trained on the available datasets (i.e., urine and serum) and validated according to the above mentioned accuracy metrics. Training, tests, and biological details on the performed experiments are reported in [27] and [40], where the here proposed framework has been used with useful results in terms of identified peptides and sample classification.

**Table 2:** Voting results for EPS-Urine dataset. In the prediction columns (*SoftVoting* and *HardVoting*), 0 is a prediction for the BPH class, and 1 stands for PCa. E.g. for *Patient Id* 141, affected by PCa (Diagnosed disease), *SoftVoting* predicted the BPH class (wrong prediction) and also *HardVoting* predicted BPH (wrong prediction)

Patient Id	Diagnosed disease (real class)	SoftVoting prediction	HardVoting prediction
121	BPH	0	0
133	BPH	0	0
39	BPH	0	0
68	BPH	0	0
84	BPH	0	0
91	BPH	1	1
1	PCa	1	1
125	PCa	1	1
126	PCa	1	1
141	PCa	0	0
17	PCa	1	1
87	PCa	1	1

**Fig. 3:**
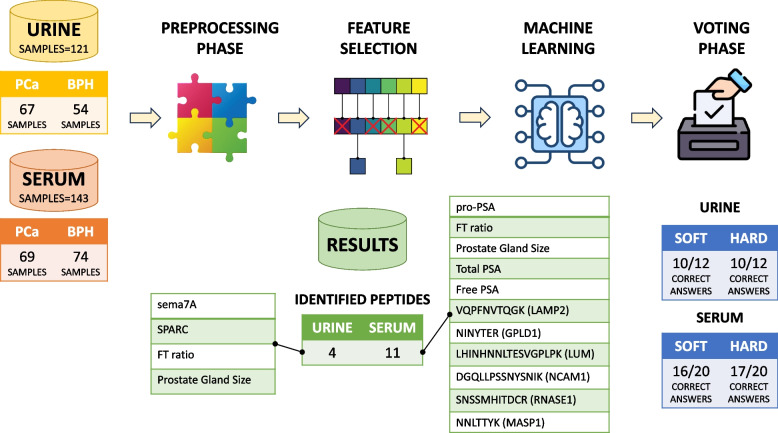
Pipeline application and description with data samples used to test the pipeline. Numerical information about data and results (i.e. peptides) are reported in the tables

Finally, the pipeline provides a voting strategy to obtain more reliable predictions on average. In our experiments, the majority (hard) voting strategy allows us to achieve the highest classification performance. For instance, in Table 2 we report two ensembling approaches used on the EPS-Urine dataset. The *Patient Id* column (same as *Id* column of Table 3) stores the information needed to identify the patient and to find its clinical data in the database. The *Diagnosed disease* column (same as the *Disease* column in Table 3) stores the disease code (classes of the machine learning task). Finally, the *SoftVoting* and *HardVoting* columns contain: (*i*) 0 if the majority (Hard Voting) or the best performing (Soft Voting) models agree on the BPH class; (*ii*) 1 if the majority (Hard Voting) or the best performing (Soft Voting) models agree on the PCa class. For instance, for patient 39, affected by BPH, both voting strategies correctly predict the class. For patient 141, affected by PCa, Soft Voting wrongly predicted the BPH class, while Hard Voting correctly predicted PCa.

**Table 3:** Example of input dataset showing a sample per row. Missing values have been represented by the *NaN* (Not a Number) literal. An excessive number of missing values will cause the elimination of the sample, while the remaining data will be statistically imputed in the preprocessing phase. Each sample is identified by its *Id*, and other relevant features are reported, e.g. the *Age* of the patient. The *Disease* feature holds the class information for each tuple (sample). The features on the right of the *Disease* column are protein expression values related to each sample

Id	Age	Prostate GlandSize	TotalPsa	FTratio	PsaFree	Disease	...	sema7a
id100	57	95.00	8.94	24.0	2.14	BPH	...	15300
id19	73	*NaN*	0.07	71.0	0.05	BPH	...	29200
id7	47	20.0	6.97	8.0	0.59	PCA	...	31800
id30	62	50.0	19.71	10.0	1.97	PCa	...	9230
id144	73	*NaN*	1.83	16.0	0.29	PCa	...	28300
id142	72	*NaN*	8.05	21.0	1.71	PCa	...	22800
...	...	...	...	...	...	...	...	...

Overall, for the EPS-Urine dataset, both the hard and soft voting strategies correctly classifies 10 out of 12 examples from the validation set (see Table 2). For the Serum dataset, the soft voting scored 16 out 20 and hard voting scored 17 out of 20.

**Fig. 4:**
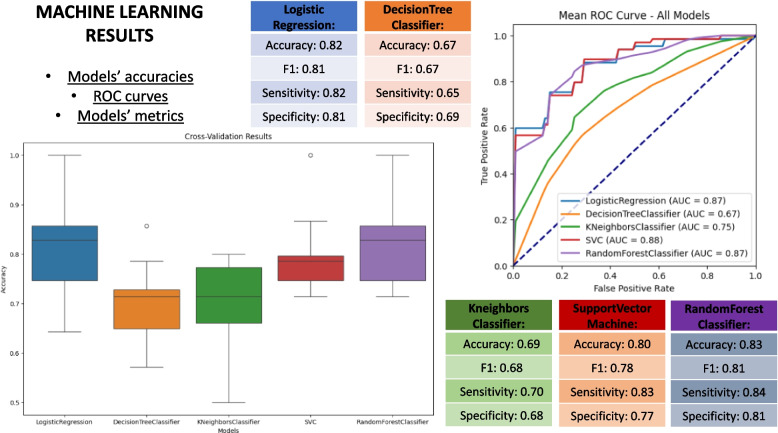
Results about accuracy metrics of the **Serum** experiment are reported in the figure with the ROC-AUC curve showing the different performances of the ML models

**Fig. 5:**
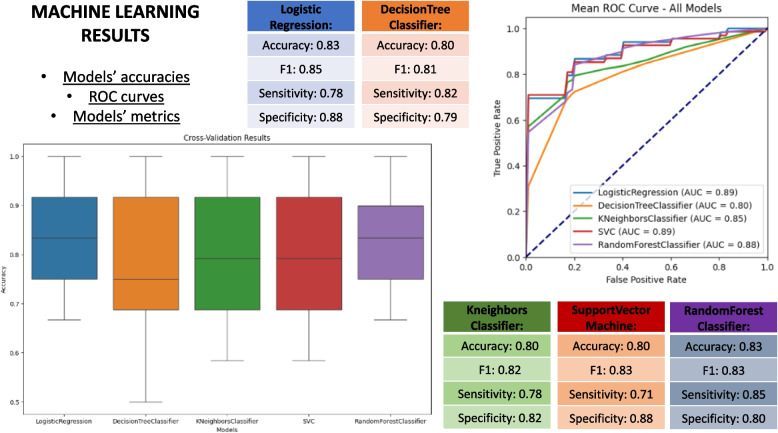
Results about accuracy metrics of the **Urine** experiment are reported in the figure with the ROC-AUC curve showing the different performances of the ML models

Both results are reported in Fig. 3, which reports the implementation of the pipeline (also described as a general architecture in Fig. 1) as applied to the above described experiments. Moreover, Figs. 4 and 5 report the accuracies of the methods in the Serum experiment, ROC-AUC curves and metrics of the five ML algorithms respectively, showing the efficacy of the proposed pipeline.

All experiments were executed in an experimental setup composed of computational resources from the Google Colab cloud environment on which the default Python 3 Runtime was used. For the Prostate serum dataset, the preprocessing phase was performed in 0.36 s, the feature selection phase in 0.4 s and the ML models training in 1.8 s. For the EPS-Urine dataset, the preprocessing phase was executed in 0.28 s, the feature selection phase in 8.43 s and the ML models training in 3.7 s. The higher execution time of the feature selection phase for the EPS-Urine dataset is due to the larger number of columns (1600 vs 39) in the training set.

## Discussion

The use of machine learning techniques to identify biomarkers has been widely applied to biological as well as clinical data [7–10], to support physicians and biologists in searching for relevant biomarkers. Deep learning techniques as well as voting mechanisms can also be used to enrich data results [24, 25]. E.g., voting mechanisms have been used in [16, 17] in order to improve proteins identification results in different contexts. Thus, using machine learning pipelines is not new in the field. For instance, in [11] ML techniques for mass spectrometry data reduction have been reported, whereas in [12, 13, 15, 41] ML techniques have been used to filter out biomarkers from prostate datasets. In [15], the authors used mass spectrometry data analysis to discover and validate prostate-derived proteins in fluids. ML models have also been used to support chronic disease-related studies. In [18] ML models have been trained to assess the potential of inflammation biomarkers in the prediction of myocardial infarction, where data has been enriched with clinical patient information. Colon cancer data classification and information extraction has been tackled in [19], by using an ensemble of ML models trained on serum biomarkers. Similarly, [20] uses ML models to identify optimal diagnostic biomarkers for non-small cell lung cancer. ML has also been used in [21] and in [22] for transcriptome profile identification related to early detection of breast cancer. Diabetes-related diseases have been studied in [23] with early detection techniques based on ML prediction models.

In this work, we present an experience of a developed and applied pipeline including ML modules which can be used to analyze biological and clinical information related to chronic diseases. We applied this pipeline for a funded research project aimed to support clinicians in studying datasets of interest for prostate related diseases. The general purpose pipeline has been applied to enrich a biological dataset with clinical data such as gland prostate dimension as an additional feature, for a large available set of patient-related data acquired during the development of the 2 years research project. Without loss of generalization, the pipeline trains ML models on the dataset proving the efficacy of the proposed method, thus that the proposed implemented pipeline can be used and integrated in different application scenarios. Thus, the pipeline goes in the direction of using ML techniques to support physicians and biologists in studying peptides and biomarkers extracted from biological samples. A voting mechanism has also been defined to choose the most suitable ML algorithm to be used on biological dataset [26]. Best model as well as ensembling model prediction results are obtained with hard (majority consensus) and soft (consensus weighted by model performance) voting approaches. The feature selection approach is used, as in several contexts in bioinformatics and health informatics, to reduce the number of features and increase the quality of the resulting models [42–45]. Feature selection methods allow the users to implement the identification of features allowing prediction performance, and a better understanding of data in machine learning or pattern recognition applications [46]. The proposed pipeline allows to include of, in the data analysis, clinical information used in the ML methods. By applying the pipeline to a real prostate cancer use case, we prove that by integrating sample clinical information (such as gland prostate size) in the analyzed biological sample the results are enriched for better clinical interpretation [27], allowing to achieve disease early predictions performance or guiding clinical procedures (such as biopsy).

Pipelines for analyzing mass spectrometry data and to identify biomarkers have been reported in many studies. In [47] a pipeline is proposed which uses multiple open-access tools, able to only process mass spectrometry data. In Weber et al. [48] a pipeline for mass spectrometry analysis dedicated to vitreous proteomics is presented, used for studying proliferative diabetic retinopathy. In [49], a data analysis pipeline for proteomics and peptidomics called DIAproteomics has been presented, able to acquire protein and peptide data from different data sources and formats, but does not consider clinical data. In [50] an open-source software suite for analysis of mass spectrometry data dedicated to translational proteomics is reported, while in [51] the IP4M platform is presented, as a modular scientific environment framework which allows biologists and domain experts in setting up complex data analysis experiments.

The here presented pipeline has a different focus concerning the above mentioned scientific environments since it is more programmer oriented. In fact, in addition to highlighting the most significant feature set for the particular task, it is able to return the trained machine learning model as a: (i) best model, which is the model showing the highest predictive performance for the task, and (ii) the integrated model, which is the ensembled model which performs better than each singular model on average. These extracted models can be used by programmers and system integrators to implement their software or even a novel pipeline or could be handy for a population-wide screening, especially in places where specialists may not be readily available.

## Conclusions

Biomarker discovery represents an important task for the automatic discrimination of biological evidence. This paper describes a software pipeline for the analysis of clinical and mass spectrometry data. The pipeline has been developed in a general purpose research project for tracking and analyzing clinical and biological datasets. We report the experience of applying it to a prostate cancer dataset, proving its efficacy in finding interesting peptides which can be considered significant features for disease prediction through biological interpretation by domain experts. The pipeline is also able to make the trained ML models available to programmers and system integrators, who can use them to build novel software platforms and pipelines for more specific tasks or domains.

## Data Availability

No datasets were generated or analysed during the current study.

## Footnotes

1 https://github.com/gtradigo/InnoprostProteomicPipeline

## References

[1] Zhou X, Mao J, Ai J, Deng Y, Roth MR, Pound C (2012). Identification of plasma lipid biomarkers for prostate cancer by lipidomics and bioinformatics. PLoS ONE..

[2] Vizza P, Pascuzzi L, Aracri F, Tavolaro E, Lambardi P, Gaspari M, et al. Prostate Cancer Disease Study by Integrating Peptides and Clinical Data. In: AAI4H@ ECAI. Amsterdam: IOS Press; 2020. p. 45–48.

[3] Pienta KJ, Esper PS (1993). Risk factors for prostate cancer. Ann Intern Med..

[4] Pierre-Victor D, Parnes HL, Andriole GL, Pinsky PF (2021). Prostate cancer incidence and mortality following a negative biopsy in a population undergoing PSA screening. Urology..

[5] White CN, Chan DW, Zhang Z (2004). Bioinformatics strategies for proteomic profiling. Clin Biochem..

[6] Petricoin EF, Ornstein DK, Paweletz CP, Ardekani A, Hackett PS, Hitt BA (2002). Serum proteomic patterns for detection of prostate cancer. J Natl Cancer Inst..

[7] Garg A, Mago V (2021). Role of machine learning in medical research: a survey. Comput Sci Rev..

[8] Mahmud M, Kaiser MS, McGinnity TM, Hussain A (2021). Deep learning in mining biological data. Cogn Comput..

[9] Li Y, Wu FX, Ngom A (2018). A review on machine learning principles for multi-view biological data integration. Brief Bioinform..

[10] Khalsan M, Machado LR, Al-Shamery ES, Ajit S, Anthony K, Mu M (2022). A survey of machine learning approaches applied to gene expression analysis for cancer prediction. IEEE Access..

[11] Fan Z, Kong F, Zhou Y, Chen Y, Dai Y. Intelligence algorithms for protein classification by mass spectrometry. BioMed Res Int. 2018;2018. 10.1155/2018/2862458 PMC6252195 30534555

[12] Taskin V, Dogan B, Ölmez T (2013). Prostate cancer classification from mass spectrometry data by using wavelet analysis and Kernel Partial Least Squares Algorithm. Int J Biosci Biochem Bioinforma..

[13] Oh JH, Lotan Y, Gurnani P, Rosenblatt KP, Gao J (2009). Prostate cancer biomarker discovery using high performance mass spectral serum profiling. Comput Methods Prog Biomed..

[14] Datta S, Pihur V. Feature selection and machine learning with mass spectrometry data. Bioinforma Methods Clin Res. 2010;593:205–29. 10.1007/978-1-60327-194-3_11 19957152

[15] Khoo A, Liu LY, Nyalwidhe JO, Semmes OJ, Vesprini D, Downes MR (2021). Proteomic discovery of non-invasive biomarkers of localized prostate cancer using mass spectrometry. Nat Rev Urol..

[16] Palopoli L, Rombo SE, Terracina G, Tradigo G, Veltri P (2009). Improving protein secondary structure predictions by prediction fusion. Inf Fusion..

[17] Theriault RL, Kaufmann M, Ren KY, Varma S, Ellis RE (2021). Metabolomics patterns of breast cancer tumors using mass spectrometry imaging. Int J CARS..

[18] Roseiro M, Henriques J, Paredes S, Rocha T, Sousa J. An interpretable machine learning approach to estimate the influence of inflammation biomarkers on cardiovascular risk assessment. Comput Methods Prog Biomed. 2023;230:107347. 10.1016/j.cmpb.2023.107347 36645940

[19] Battista A, Battista RA, Battista F, Iovane G, Landi RE (2021). BH-index: a predictive system based on serum biomarkers and ensemble learning for early colorectal cancer diagnosis in mass screening. Comput Methods Prog Biomed..

[20] Wang F, Su Q, Li C (2022). Identidication of novel biomarkers in non-small cell lung cancer using machine learning. Sci Rep..

[21] Taghizadeh E, Heydarheydari S, Saberi A, JafarpoorNesheli S, Rezaeijo SM (2022). Breast cancer prediction with transcriptome profiling using feature selection and machine learning methods. BMC Bioinformatics..

[22] Botlagunta M, Botlagunta MD, Myneni MB, Lakshmi D, Nayyar A, Gullapalli JS (2023). Classification and diagnostic prediction of breast cancer metastasis on clinical data using machine learning algorithms. Sci Rep..

[23] Kopitar L, Kocbek P, Cilar L, Sheikh A, Stiglic G (2020). Early detection of type 2 diabetes mellitus using machine learning-based prediction models. Sci Rep..

[24] Srivastava S, Soman S, Rai A, Srivastava PK. Deep learning for health informatics: recent trends and future directions. In: 2017 International Conference on Advances in Computing, Communications and Informatics (ICACCI). IEEE; 2017. p. 1665–1670.

[25] Callahan A, Shah NH. Machine learning in healthcare. In: Key Advances in Clinical Informatics. Elsevier; 2017. p. 279–291.

[26] Paul TK, Iba H (2008). Prediction of cancer class with majority voting genetic programming classifier using gene expression data. IEEE/ACM Trans Comput Biol Bioinforma..

[27] Prestagiacomo L, Tradigo G, Aracri F, Gabriele C, Rota MA, Alba S, et al. Data-Independent Acquisition Mass Spectrometry of EPS-urine coupled to Machine Learning: a predictive model for prostate cancer. ACS Omega; 2023. 10.1021/acsomega.2c05487 PMC9948177 36844540

[28] Gabriele C, Aracri F, Prestagiacomo LE, Rota MA, Alba S, Tradigo G (2023). Development of a predictive model to distinguish prostate cancer from benign prostatic hyperplasia by integrating serum glycoproteomics and clinical variables. Clin Proteomics..

[29] Beg M, Taka J, Kluyver T, Konovalov A, Ragan-Kelley M, Thiéry NM (2021). Using Jupyter for reproducible scientific workflows. Comput Sci Eng..

[30] Mukaka MM (2012). A guide to appropriate use of correlation coefficient in medical research. Malawi Med J..

[31] Tallarida RJ, Murray RB. Chi-square test. In: Manual of pharmacologic calculations. Springer; 1987. p. 140–142.

[32] Vanjimalar S, Ramyachitra D, Manikandan P. A review on feature selection techniques for gene expression data. In: 2018 IEEE International Conference on Computational Intelligence and Computing Research (ICCIC). IEEE; 2018. p. 1–4.

[33] Speiser JL, Miller ME, Tooze J, Ip E (2019). A comparison of random forest variable selection methods for classification prediction modeling. Expert Syst Appl..

[34] Christodoulou E, Ma J, Collins GS, Steyerberg EW, Verbakel JY, Van Calster B (2019). A systematic review shows no performance benefit of machine learning over logistic regression for clinical prediction models. J Clin Epidemiol..

[35] Huang HC, Zheng S, Zhao Z (2010). Application of Pearson correlation coefficient (PCC) and Kolmogorov-Smirnov distance (KSD) metrics to identify disease-specific biomarker genes. BMC Bioinformatics..

[36] Wang L, Jiang Z, Sui M, Shen J, Xu C, Fan W (2009). The potential biomarkers in predicting pathologic response of breast cancer to three different chemotherapy regimens: a case control study. BMC Cancer..

[37] Lv Y, Wang Y, Tan Y, Du W, Liu K, Wang H. Pancreatic cancer biomarker detection using recursive feature elimination based on Support Vector Machine and large margin distribution machine. 4th International Conference on Systems and Informatics (ICSAI). New York: IEEE; 2017. p. 1450–1455.

[38] Ram M, Najafi A, Shakeri MT (2017). Classification and biomarker genes selection for cancer gene expression data using random forest. Iran J Pathol..

[39] Aggarwal CC, et al. Data mining: the textbook, vol 1. Springer; 2015.

[40] Gabriele C, Aracri F, Prestagiacomo LE, Rota MA, Alba S, Tradigo G, et al. Development of a predictive model of prostate cancer: integration of a panel of formerly N-linked glycopeptides and clinical variables for serum testing. 2022. 10.21203/rs.3.rs-2036305/v1.

[41] Cannataro M, Guzzi PH, Mazza T, Tradigo G, Veltri P (2007). Using ontologies for preprocessing and mining spectra data on the Grid. Futur Gener Comput Syst..

[42] Din S, Paul A, Guizani N, Ahmed SH, Khan M, Rathore MM. Features selection model for internet of e-health things using big data. In: GLOBECOM 2017-2017 IEEE Global Communications Conference. IEEE; 2017. p. 1–7.

[43] Naheed N, Shaheen M, Khan SA, Alawairdhi M, Khan MA (2020). Importance of features selection, attributes selection, challenges and future directions for medical imaging data: a review. Comput Model Eng Sci..

[44] Goh WWB, Wong L (2019). Advanced bioinformatics methods for practical applications in proteomics. Brief Bioinform..

[45] Gallo Cantafio ME, Grillone K, Caracciolo D, Scionti F, Arbitrio M, Barbieri V (2018). From single level analysis to multi-omics integrative approaches: a powerful strategy towards the precision oncology. High-throughput..

[46] Chandrashekar G, Sahin F (2014). A survey on feature selection methods. Comput Electr Eng..

[47] Malm EK, Srivastava V, Sundqvist G, Bulone V (2014). APP: an Automated Proteomics Pipeline for the analysis of mass spectrometry data based on multiple open access tools. BMC Bioinformatics..

[48] Weber SR, Zhao Y, Ma J, Gates C, da Veiga Leprevost F, Basrur V (2021). A validated analysis pipeline for mass spectrometry-based vitreous proteomics: new insights into proliferative diabetic retinopathy. Clin Proteomics..

[49] Bichmann L, Gupta S, Rosenberger G, Kuchenbecker L, Sachsenberg T, Ewels P (2021). DIAproteomics: a multifunctional data analysis pipeline for data-independent acquisition proteomics and peptidomics. J Proteome Res..

[50] Keller A, Shteynberg D. Software pipeline and data analysis for MS/MS proteomics: the trans-proteomic pipeline. Bioinforma Comp Proteomics. 2011;694:169–89. 10.1007/978-1-60761-977-2_12 21082435

[51] Liang D, Liu Q, Zhou K, Jia W, Xie G, Chen T (2020). IP4M: an integrated platform for mass spectrometry-based metabolomics data mining. BMC Bioinformatics..

